# Detecting plasma hsa_circ_0061276 in patients with gastric cancer by reverse transcription-digital polymerase chain reaction

**DOI:** 10.3389/fonc.2022.1042248

**Published:** 2022-12-23

**Authors:** Yao Ruan, Zhe Li, Yaoyao Xie, Weiliang Sun, Junming Guo

**Affiliations:** ^1^ Department of Gastrointestinal Surgery, The Affiliated People’s Hospital of Ningbo University, Ningbo, China; ^2^ Department of Biochemistry and Molecular Biology, and Zhejiang Key Laboratory of Pathophysiology, School of Basic Medical Sciences, School of Medicine, Ningbo University, Ningbo, China; ^3^ Institute of Gastrointestinal Tumor of Ningbo University, Ningbo, China

**Keywords:** circular RNA, reverse transcription digital PCR (RT-dPCR), tumor markers, quantitative detection, miRNA sponge, hsa_circ_0061276, hsa-miR-7705

## Abstract

**Background:**

The role of circular RNAs (circRNAs) in the occurrence of gastric cancer is still unclear. Therefore, the diagnostic value and mechanisms underlying hsa_circ_0061276 in the occurrence of gastric cancer were explored.

**Methods:**

Reverse transcription-droplet digital polymerase chain reaction was used to detect the copy number of hsa_circ_0061276 in plasma from healthy individuals, as well as from patients with gastric precancerous lesions or early-stage or advanced gastric cancer. Plasmids overexpressing or knocking down hsa_circ_0061276 expression were transfected into gastric cancer cells. The effects on the growth, migration, and cell cycle distribution of gastric cancer cells were then analyzed. Finally, miRanda and RNAhybrid were used to explore the binding sites between hsa_circ_0061276 and microRNAs (miRNAs). A double luciferase reporter gene assay was used to confirm the miRNA sponge effect.

**Results:**

The results show that plasma hsa_circ_0061276 copy number showed a trend of a gradual decrease when comparing healthy controls to the early cancer group and advanced gastric cancer group. Overexpression of hsa_circ_0061276 inhibited the growth and migration of gastric cancer cells. Through bioinformatic analyses combined with cellular experiments, it was found that hsa_circ_0061276 inhibited the growth of gastric cancer by binding to hsa-miR-7705.

**Conclusion:**

Hsa_circ_0061276 may be a new biomarker for gastric cancer. The tumor suppressor role of hsa_circ_0061276 on gastric cancer likely occurs through a sponge effect on miRNAs such as hsa-miR-7705.

## Introduction

Gastric cancer is the fifth most common cancer worldwide (1, 2). Since early-stage gastric cancer has no specific clinical manifestations, the best time for the treatment of patients with gastric cancer is often missed by the time of diagnosis (3). The 5-year survival rate of advanced gastric cancer patients is less than 10%. However, if gastric cancer is diagnosed early, the 5-year survival rate can be up to 90% (4). Therefore, early detection and treatment is extremely important for gastric cancer patients. At present, gastroscopies can be used to diagnose gastric cancer early in the clinic (5). However, during the endoscopy process, patients will experience varying degrees of discomfort such as nausea and vomiting. As a result, it is necessary to establish a non-invasive screening method for gastric cancer.

Circular RNAs (circRNAs) have received widespread attention in recent years (6–8). Their unique circular structure results in their high stability. circRNAs commonly exist in various human body fluids and exosomes, offering the potential of using circRNAs as biomarkers of gastric cancer (9, 10). Many researchers have elucidated the multiple functions of circRNAs, such as competitively binding to proteins and microRNAs (miRNAs), participating in protein coding, and regulating gene transcription (11–14). The miRNA sponge function is the most studied (11–14).

The droplet digital polymerase chain reaction (ddPCR) belongs to the third-generation PCR technology (15). It uses water-in-oil technology to divide the reaction system into tens of thousands of droplets, which can be used for the absolute quantitative detection of circRNAs (16). The occurrence of gastric cancer is a gradual evolutionary process (17). Hsa_circ_0061276 is a gastric cancer-associated circRNA (18). In this study, the reverse transcription-droplet digital polymerase chain reaction (RT-ddPCR) was first used to analyze the changes in plasma hsa_circ_0061276 levels among healthy people, patients with precancerous lesions (dysplasia and intestinal metaplasia), and early gastric cancer patients. Then, to clarify the possible mechanism underlying hsa_circ_0061276 in the occurrence of gastric cancer, cell proliferation, migration, and cell cycle distribution of gastric cancer cells were studied by altering hsa_circ_0061276 expression through plasmid transfections of gastric cells. The results show, for the first time, that hsa_circ_0061276 inhibits the growth of gastric cancer cells through a miRNA sponge effect.

## Materials and methods

### Plasma specimens

Fasting plasma collected from healthy individuals who had a physical examination in the Affiliated Hospital of Ningbo University School of Medicine in November 2019 were used as controls. The fasting plasma of patients with gastric precancerous lesions or early-stage or advanced gastric cancer (**Supplementary Table 1**) was obtained from The Affiliated Hospital, The Affiliated Ningbo No. 1 Hospital, and The Affiliated People’s Hospital at Ningbo University.

All patients had undergone a gastroscopy and histopathological diagnosis. Pairs of gastric cancer tissues and para-cancerous tissues were obtained from the above hospitals. Clinical data were provided by professional pathologists and clinicians. The blood of patients with gastric cancer was collected before surgery and prior to any treatment. Blood was collected in ethylenediamine tetraacetic acid (EDTA) tubes (BD Biosciences, Franklin Lakes, NJ, USA). All blood samples were first centrifuged at 4,000 RPM for 10 min, and then the upper layer of light-yellow plasma was isolated and stored at −80°C until further use. This study was approved by the Medical Ethics Committee of Ningbo University (IRB No. 2017022701). Written informed consent was obtained from all participants.

### Cell culture

The normal gastric mucosal epithelial cell line GES-1 was obtained from Beijing Beina Chuanglian Institute of Biotechnology, China. Gastric cancer cell lines, HGC-27 and AGS, were obtained from the Cell Resource Center, Chinese Academy of Sciences (Shanghai, China). Human embryonic kidney (HEK) 293T cells were provided by Professor Zhaohui Gong, School of Medicine, Ningbo University. Roswell Park Memorial Institute (RPMI) and Dulbecco’s Modified Eagle’s Medium (DMEM) (HyClone, Los Angeles, CA, USA) containing 1% penicillin/streptomycin (Life Technologies, Carlsbad, CA, USA) and 12% fetal bovine serum (Gibco, Grand Island, NY, USA) were used. The cell incubator was set to 37°C and 5% CO_2_.

### Extraction of total RNA

Following the manufacturer’s instructions for total RNA extraction, TRIzol LS or TRIzol (Invitrogen, Carlsbad, CA, USA) was added to the plasma and tissues or cells, respectively. The SmartSpec Plus spectrophotometer (Denovix, Hercules, CA, USA) was used to determine RNA purity and concentration. RNA was then stored at −80°C.

### RT-PCR

Total RNA was reverse transcribed into cDNA by the GoScript Reverse Transcription (RT) System (Promega, Madison, WI, USA) and the Hairpin-it™ miRNAs quantitative RT-PCR Kit (GenePharma, Shanghai, China).

To detect hsa_circ_0061276 and hsa-miR-7705 levels in cells and tissues, qRT-PCR was performed on the Mx3005P PCR system (Stratagene, Palo Alto, CA, USA) using GoTaq qPCR Master Mix (Promega). U6 small nuclear RNA (snRNA) and glyceraldehyde-3-phosphate dehydrogenase (GAPDH) mRNA were used as references for relative expression of hsa-miR-7705 and hsa_circ_0061276, respectively. For the detection of hsa_circ_0061276 and GAPDH mRNA, a pre-denaturation step at 95°C for 10 min was performed. Then, 40 cycles of denaturation at 94°C for 15 s, annealing at 57°C for 30 s, and extension at 72°C for 30 s were performed. For the detection of hsa-miR-7705 and U6 snRNA, a pre-denaturation step at 95°C for 3 min was performed, followed by 40 cycles of denaturation at 95°C for 12 s, and annealing and extending at 62°C for 40 s. The sequences for RT-PCR are shown in **Supplementary Table 2**.

### RT-ddPCR

A PCR system using 2× QX200 EvaGreen ddPCR Supermix (Bio-Rad, Hercules, CA, USA) with a total volume of 20 µl was first prepared and then subjected to vortex oscillation and centrifugation to remove bubbles.

For droplet generation, the micro-drop generation card was put into the holder in the notch direction. Then, the total reaction system was transferred to the middle row hole of the microliter generation card. Finally, 70 μl of EvaGreen Droplet Generation Oil (Bio-Rad) was added to the lower row hole. A piece of rubber pad was used to cover the holder, and then the micro-drop generation reaction was performed using a QX200™ Droplet Generator (Bio-Rad).

After micro-droplet generation, the upper row of micro-droplets (about 40 µl) was transferred to a 96-well plate. Then, the 96-well plate was covered with a film and put into a film-sealing instrument (Bio-Rad). Next, the film was sealed at 180°C. The 96-well plate was taken out and PCR was performed using a T100™ Thermal Cycler (Bio-Rad). For PCR, a pre-denaturation step was performed at 95°C for 5 min. Then, 40 cycles of denaturation at 95°C for 30 s and annealing/extending at 57°C for 60 s was performed. Finally, the amplified 96-well plate was detected on a QX200™ Droplet Reader (Bio-Rad).

### RNA stability test

Actinomycin D (APE×BIO, Houston, TX, USA) was used to study the stability of circRNA by comparing the half-life of linear RNA and circRNA. Five time points at 0 h, 4 h, 8 h, 12 h, and 24 h were selected. No treatment was performed at 0 h, and 10 μg of actinomycin D solution was added to each well of the remaining four groups. Total RNA was first extracted at 0 h, 4 h, 8 h, 12 h, and 24 h, and then the levels of hsa_circ_0061276 and its corresponding linear transcript, nuclear receptor interacting protein 1 (NRIP1) mRNA, were measured using qRT-PCR.

### Up- or downregulating hsa_circ_0061276 expression

To upregulate or downregulate hsa_circ_0061276 expression in GES-1, AGS, and HGC-27 cells, 5 µl of Lipofectamine 2000 (Life Technologies) and 2.5 µg of hsa_circ_0061276 overexpression plasmid (Geneseed, Guangzhou, China) or downregulation plasmid with short hairpin RNAs (shRNA) against hsa_circ_0061276 were added into 200 μl of Opti-MEM low serum culture medium (Gibco). After mixing, the medium was added into a six-well plate with cultured cells and then incubated for 24–48 h. Three shRNA plasmids (sh1, sh2, and sh3) targeting the joint site of hsa_circ_0061276 were provided by Geneseed. Screening experiments revealed that sh1 and sh3 were effective, and these shRNAs were then used in the following experiments. For upregulation or downregulation experiments, the respective empty plasmids were used as a control. The vector structures are shown in **Supplementary Figure 1**.

### Cell proliferation and colony formation assays

Cell proliferation after transfection with hsa_circ_0061276 overexpressing plasmids or hsa_circ_0061276 shRNA plasmids (Geneseed) was detected using Cell Counting Kit-8 (CCK-8) (Dojindo, Tokyo, Japan). After culturing for 24 h, 48 h, 72 h, and 96 h, 10 μl/well of CCK-8 reagent was added to the cultures in the dark, and then the cultures were incubated for 3 h. The SpectraMax M5 reader (Molecular Devices, Silicon Valley, CA, USA) was used to detect the plate optical density (OD) at 450 nm.

Forty-eight hours post-transfection with hsa_circ_0061276 overexpressing plasmids or shRNA plasmids (Geneseed), colony formation experiments were performed as previously reported (19). Forty-eight hours after cell transfection, cells were inoculated into a six-well plate (1,000 cells per well). After culturing cells for 10 days, colonies were first stained with crystal violet (Solarbio, Beijing, China) and then counted using Photoshop software (Adobe, San Jose, CA, USA).

### Transwell migration assay

Cell migration ability was detected using a Transwell assay. Cells were first transfected with hsa_circ_0061276 overexpressing plasmids or shRNA plasmids (Geneseed) for 48 h. Cells were then seeded in a small chamber (40,000 cells per well) (Corning Inc. Corning, NY, USA). A total of 750 µl of 12% RPMI 1640 medium (HyClone) was added to the lower chamber. After culturing cells for 48 h, cells were fixed with 4% paraformaldehyde. Then, cells were stained with 0.1% crystal violet. Finally, cells in three random fields were counted.

### Cell cycle assay

To study the effect of hsa_circ_0061276 on the cell cycle, gastric cancer cells were first transfected with hsa_circ_0061276 overexpressing plasmids or shRNA plasmids (Geneseed) for 48 h. The distribution of the cell cycle was then measured using flow cytometry (BD Bioscience) following a previously published protocol (4). Briefly, the transfected and starved cells were washed twice with phosphate buffered saline (PBS) and centrifuged at 1,000 RPM for 4 min. Cells were then fixed in anhydrous ethanol at a ratio of 1:3 at −20°C for 24 h. Cells were then centrifuged at 1,500 RPM for 5 min. Next, 1 ml of DNA-stabilizing solution was added, and cells were incubated at room temperature in the dark for 30 min. Finally, cell cycle distribution was analyzed. All experiments were repeated three times.

### Cytoplasmic and nuclear RNA purification

The cytoplasmic and nuclear RNA from 1 × 10^7^ HEK 293T, HGC-27, and AGS cells were first purified. Briefly, 400 µl of extracted and pre-cooled Cell Fractionation Buffer (Thermo Fisher Scientific, New York, NY, USA) was first added to the cells and then the cells were incubated on ice for 10 min. Cells were centrifuged at 500 × *g* for 5 min (4°C). The cytoplasmic part (supernatant) was transferred to a 1.5-ml RNase-free centrifuge tube. The remaining part was the nuclear component.

After the supernatant was transferred to another tube, 400 µl of pre-cooled Cell Fractionation Buffer was added. Then, to remove the supernatant, the tube was centrifuged at 50 × *g* for 1 min (4°C). Finally, 400 µl of Cell Disruption Buffer and 400 µl 2× Lysis/Binding Solution was added to the sediment and mixed well at room temperature. Next, 400 µl of absolute ethanol was added and the samples were centrifuged at 14,000 RPM for 1 min at room temperature. Then, 700 µl of Wash Solution was added to the sample and then samples were centrifuged at 14,000 RPM for 1 min. The supernatant was then discarded. After repeating this wash step twice, 40 μl of Elution Solution (preheated to 95°C) was added and the samples were centrifuged at 14,000 RPM at room temperature for 30 s. Finally, cytoplasmic and nuclear RNA were reverse transcribed into cDNA following the manufacturer’s protocol (ThermoFisher Scientific, New York, NY, USA).

### circRNA–miRNA interaction screening and double luciferase reporter gene assay

To explore the mechanism of the effect of hsa_circ_0061276 on the growth of gastric cancer, circRNA–miRNA interaction screening software, including RNAhybrid (https://bibiserv.cebitec.uni-bielefeld.de/applications/rnahybrid/) and miRanda (www.miranda.org), was used to explore the possible binding between hsa_circ_0061276 and miRNAs. Combined with the ENCORI (The Encyclopedia of RNA Interactomes, http://starbase.sysu.edu.cn/index.php) database, target miRNAs were identified. To further confirm the binding sites between hsa_circ_0061276 and miRNA, a double luciferase reporter gene assay was used. Briefly, four groups of co-transfections, namely, miRNA mimics negative control (NC) + hsa_circ_0061276 wild type (WT), miRNA mimics + hsa_circ_0061276 WT, miRNA mimics NC + hsa_circ_0061276 mutation (MUT), and miRNA mimics + hsa_circ_0061276 MUT, were prepared. A total of 20 µM of miRNA mimics or mimics NC (GenePharma, Shanghai, China) and 2.5 µg of dual luciferase carrier WT/MUT (GenePharma) in 2.5 µl, 4 µl of Lipofectamine 2000 (Life Technologies), and 100 µl of Opti-MEM low serum medium (Gibco) were used. After incubating cells with the mixtures for 24–48 h, a Dual-Luciferase^®^ Reporter Assay System (Promega) was used.

### Statistical analysis

Statistical Product and Service Solutions (SPSS) V.19.0 (IBM, Almont, NY, USA) and GraphPad 8.0 (GraphPad Software Inc., San Diego, CA, USA) software were used for data analysis and visualization. The analysis methods, paired sample *t*-test (two-tailed), two independent sample *t*-test (two-tailed), and analysis of variance (ANOVA), were selected to analyze data represented as the mean ± standard deviation (SD). If *p* < 0.05, the difference among various groups was considered statistically significant.

## Results

### Stability test of hsa_circ_0061276 in cells and plasma

Actinomycin D interferes with transcription by inhibiting RNA polymerase activity. Therefore, actinomycin D was used to measure RNA stability. The results show that the levels of hsa_circ_0061276 were higher than its linear RNA when gastric cancer cells were treated with actinomycin D (**Supplementary Figure 2A**), indicating that hsa_circ_0061276 was more stable than its parental linear RNA.

Next, repeatability testing on hsa_circ_0061276 levels in 15 randomly selected healthy human plasma samples was performed. The results show that there were no statistically significant differences between the two groups (**Supplementary Figure 2B**). Finally, 72 randomly selected fasting plasma samples from healthy donors were divided into 12 groups and placed at different temperatures for different times: room temperature, 4°C, and −20°C for 0 h, 2 h, 6 h, and 12 h. The results show that there were no differences between each time point (**Supplementary Figure 2C**), indicating that these storage conditions met the requirements of clinical testing.

### Absolute quantitative detection of plasma hsa_circ_0061276 using RT-ddPCR

According to the pathological process of gastric cancer, the copy number of plasma hsa_circ_0061276 from four groups was detected by RT-ddPCR. Compared with the healthy group, the copy number of plasma hsa_circ_0061276 was significantly reduced in the three other groups (**Supplementary Figure 3**). The copy number of plasma hsa_circ_0061276 showed a gradual decreasing trend from healthy people to early gastric cancer and to advanced gastric cancer.

The area under the receiver operating characteristic (ROC) curve for all groups was greater than 0.5 (**Supplementary Table 3**). The area under the ROC curve (AUC) between healthy people and the advanced gastric cancer group was the largest, with an AUC of 0.7380 and a specificity of 92.22%, indicating that hsa_circ_0061276 in plasma can be used to distinguish patients with advanced gastric cancer from healthy people. The AUC between healthy people and the early cancer group was 0.6978, with 90.00% specificity, indicating that plasma hsa_circ_0061276 is a good potential biomarker for the screening of early gastric cancer.

### The expression of hsa_circ_0061276 in gastric cancer cell lines

To explore the baseline hsa_circ_0061276 expression levels in gastric cancer, the relative expression of hsa_circ_0061276 in the normal gastric mucosal epithelial GES-1 cell line and in gastric cancer HGC-27 and AGS cells was detected by qRT-PCR. The results show that, compared with GES-1, hsa_circ_0061276 showed reduced expression in both HGC-27 and AGS cells (**Supplementary Figure 4**).

### The tumor suppressor role of hsa_circ_0061276 in gastric cancer

**Figure 1:**
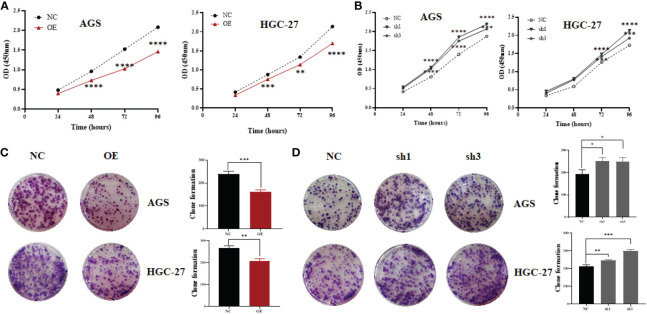
Proliferation of gastric cancer cells after upregulating or downregulating the expression of hsa_circ_0061276. **(A)** Growth curve of AGS and HGC-27 cells after upregulating hsa_circ_0061276; OE, overexpression plasmid; NC, negative control plasmid. **p < 0.01, ***p < 0.001, ****p < 0.0001; *n* = 3. **(B)** Growth curve of AGS and HGC-27 cells after downregulation of hsa_circ_0061276; NC, negative control plasmid; sh1 and sh3, short hairpin RNA plasmids. **p < 0.01, ***p < 0.001, ****p < 0.0001; *n* = 3. **(C)** Representative clones of gastric cancer cells after upregulating hsa_circ_0061276 (left) and the statistical analysis results (right). OE, overexpression plasmid; NC, negative control plasmid. **p < 0.01, ***p < 0.001; *n* = 3. **(D)** Representative clones of gastric cancer cells after downregulating hsa_circ_0061276 (left) and the statistical analysis results (right). NC, negative control plasmid; sh1 and sh3, short hairpin RNA plasmids; *p < 0.05, **p < 0.01, ***p < 0.001; *n* = 3. Independent samples *t*-test (two-tailed) for all.

To explore the biological role of hsa_circ_0061276 in gastric cancer, loss-of-function and gain-of-function approaches were used. The results of CCK-8 experiments indicate that when hsa_circ_0061276 was overexpressed, the growth of gastric cancer HGC-27 and AGS cells was significantly inhibited (**Figure 1A**). On the other hand, when hsa_circ_0061276 was downregulated, the growth of gastric cancer AGS and HGC-27 cells was significantly increased, and sh1 and sh3 showed a similar effect (**Figure 1B**). Colony formation assay experiments demonstrated that when hsa_circ_0061276 was upregulated, there were fewer AGS and HGC-27 cell clones compared with the control group (**Figure 1C**). When hsa_circ_0061276 was downregulated, the numbers of clones of the two gastric cancer cells increased compared with the control group (**Figure 1D**).

**Figure 2:**
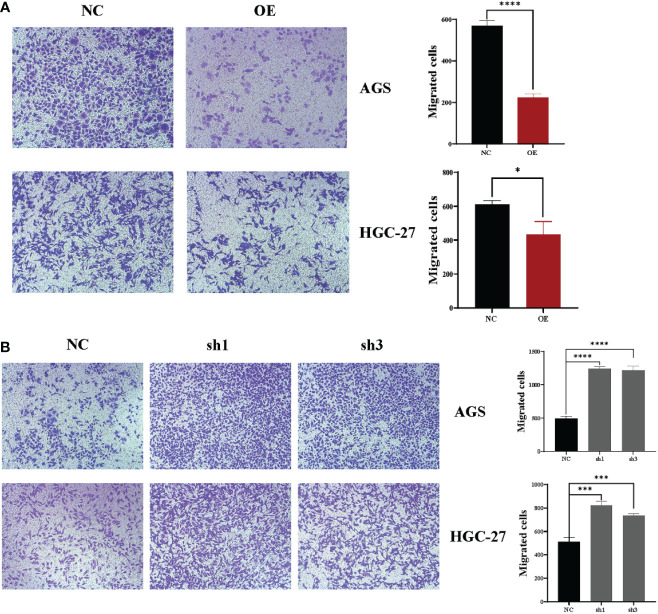
Changes in the migration ability of gastric cancer cells after upregulation and downregulation of hsa_circ_0061276 in a Transwell migration assay. **(A)** Representative results of the effect of hsa_circ_0061276 overexpression on the migration ability of cells (left, 10×) and the statistical analysis results (right). OE, overexpression plasmid; NC, negative control plasmid. *p < 0.05, ****p < 0.0001; *n* = 3. **(B)** Representative results of the effect of hsa_circ_0061276 downregulation on the migration ability (left, 10×) and the statistical analysis results (right). NC, negative control plasmid; sh1 and sh3, short hairpin RNA plasmids; ***p < 0.001, ****p < 0.0001; *n* = 3. Independent samples *t*-test (two-tailed) for A and B.

The next step was to use Transwell experiments to analyze the effect of hsa_circ_0061276 on the migration ability of cells. It was found that overexpression of hsa_circ_0061276 suppressed the migration of gastric cancer cells (**Figure 2A**), while downregulation promoted cell migration (**Figure 2B**).

**Figure 3:**
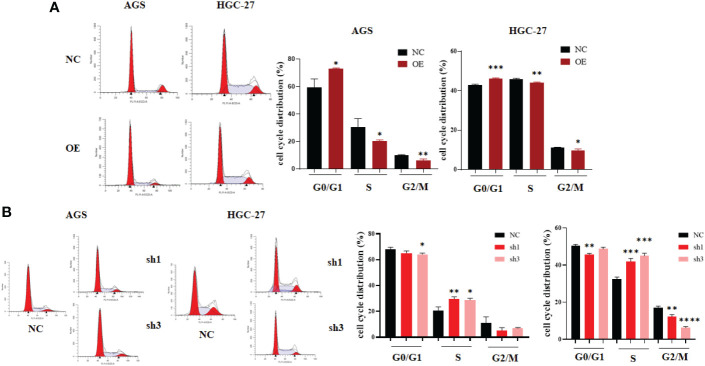
Cell cycle distribution of gastric cancer cells after upregulation and downregulation of hsa_circ_0061276. **(A)** Representative data of cell cycle distribution after upregulation of hsa_circ_0061276 (left) and the statistical analysis results (right). OE, expressed plasmid; NC, negative control plasmid. *p < 0.05, **p < 0.01, ***p < 0.001, *n* = 3. **(B)** Representative cell cycle distribution after downregulation of hsa_circ_0061276 (left) and the statistical analysis results (right). NC, negative control plasmid; sh1 and sh3, short hairpin RNA plasmids; *p < 0.05, **p < 0.01, ***p < 0.001, ****p < 0.0001, *n* = 3. Independent samples *t*-test (two-tailed) for A and B.

Finally, flow cytometry was used to analyze the change in cell cycle distribution caused by hsa_circ_0061276. Both gastric cancer cell lines were arrested at the G0/G1 phase by upregulation of hsa_circ_0061276 (**Figure 3A**). When hsa_circ_0061276 was downregulated, cells were arrested in S phase (**Figure 3B**), indicating that hsa_circ_0061276 promotes the growth of cancer cells.

### miRNA sponge function of hsa_circ_0061276

**Figure 4:**
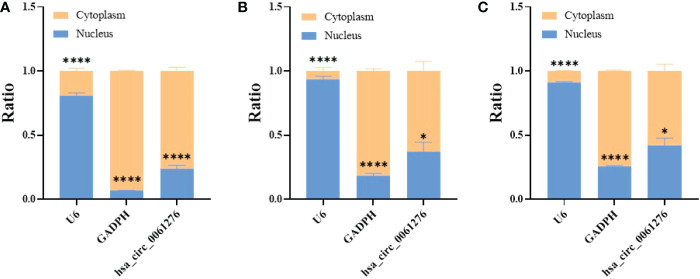
Distribution of hsa_circ_0061276 in cell fractions. qRT-PCR was used to detect the level of hsa_circ_0061276 in the nuclear and cytoplasmic fractions of 293T **(A)**, AGS **(B)**, and HGC-27 **(C)** cell lines. *p < 0.05, ****p < 0.0001, *n* = 3. Independent samples *t*-test (two-tailed).

To understand the possible mechanisms underlying hsa_circ_0061276 on the growth of gastric cancer, the nuclear and cytoplasmic RNA of HEK 293T cells and gastric cancer AGS and HGC-27 cells were extracted. U6 snRNA and GAPDH mRNA were used as the markers of the nuclear and cytoplasmic components, respectively (20). It was found that hsa_circ_0061276 was mainly expressed in the cytoplasm (**Figure 4**). Therefore, it can be considered that hsa_circ_0061276 primarily plays its role in the cytoplasm.

Since hsa_circ_0061276 largely exists in the cytoplasm, and the miRNA sponge functions take place in the cytoplasm (20), the possible interaction between hsa_circ_0061276 and miRNAs was explored. RNAhybrid and miRanda were used to predict the possible target miRNAs of hsa_circ_0061276. Then, using the ENCORI database, the expression of miRNA in gastric cancer was further investigated. Hsa-miR-6504-5p, hsa-miR-7705, and hsa-miR-7974 were highly expressed in gastric cancer cells (**Supplementary Figure 5**) and were selected for further analysis.

**Figure 5:**
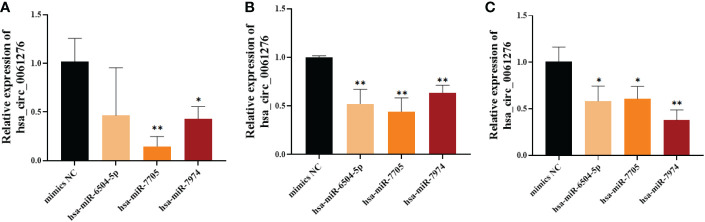
The expression of hsa_circ_0061276 in gastric cancer cells after transfection with miRNA mimics. **(A)** Twenty-five picomolar miRNA mimics. **(B)** Fifty picomolar miRNA mimics. **(C)** Seventy-five picomolar miRNA mimics. *p < 0.05, **p < 0.01; *n* = 3. Independent samples *t*-test (two-tailed).

To further verify the relationship between the binding of hsa_circ_0061276 with hsa-miR-6504-5p, hsa-miR-7705, or hsa-miR-7974, miRNA mimics were transfected into HGC-27 cells (25 pM, 50 pM, and 75 pM). The expression of hsa_circ_0061276 in gastric cancer cells was downregulated by the above miRNA mimics (**Figure 5**). Among these miRNA mimics, transfection with 25 pM hsa-miR-7705 had the greatest effect. As a result, hsa-miR-7705 was selected for further study.

**Figure 6:**
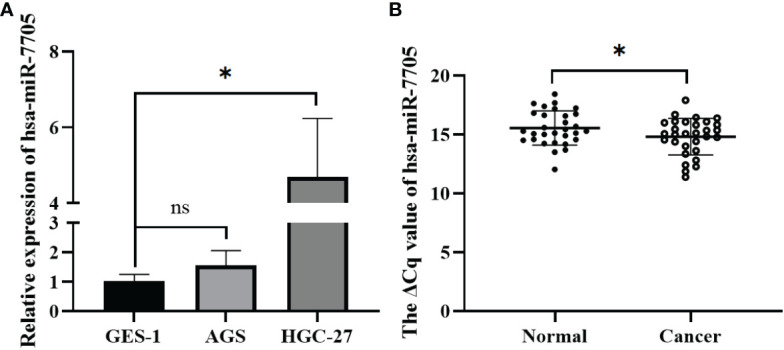
Expression of hsa-miR-7705 in gastric cancer cells and gastric cancer tissues. **(A)** Expression of has-miR-7705 in gastric cancer cells. Independent samples *t*-test (two-tailed); ns, not significant; *p < 0.05, *n* = 3. **(B)** Expression of hsa-miR-7705 in gastric cancer tissues. Paired sample *t*-test (two-tailed); *p < 0.05, *n* = 30.

To confirm the biological role of hsa-miR-7705 in gastric cancer, we measured its expression level in gastric cancer cells and gastric cancer tissues. It was found that hsa-miR-7705 was relatively highly expressed in gastric cancer cells and tissues (**Figure 6**), which is the opposite of what was measured for the expression levels of hsa_circ_0061276 in gastric cancer cells (**Supplementary Figure 4**).

**Figure 7:**
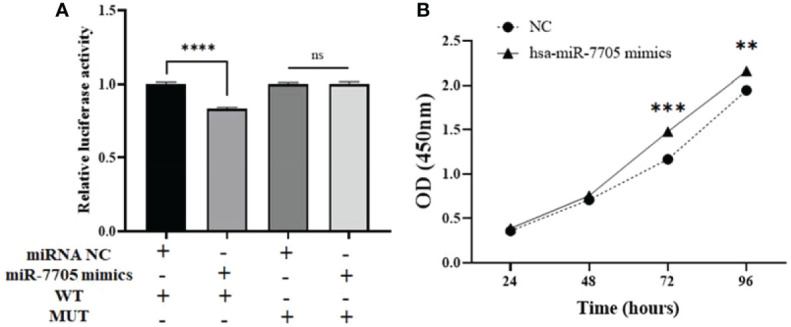
miRNA sponge function of hsa_circ_0061276. **(A)** Results of dual luciferase reporter gene experiment. Ns, not significant; ****p < 0.0001, *n* = 3. **(B)** Proliferation of HGC-27 cells after transfection with hsa-miR-7705 mimics. **p < 0.01, ***p < 0.001; *n* = 3. Independent samples *t*-test (two-tailed) for A and B.

To further confirm the binding sites between hsa_circ_0061276 and hsa-miR-7705, MUT and WT hsa_circ_0061276 plasmids were constructed for dual luciferase reporter gene detection. The luciferase activity with the MUT remained unchanged, while that of the WT was decreased (**Figure 7A**). After transfection of hsa-miR-7705 mimics into HGC-27 cells, CCK-8 proliferation experiments showed that hsa-miR-7705 mimics increased the proliferation of gastric cancer cells (**Figure 7B**). These results indicate that hsa_circ_0061276 had miRNA sponge functions and that hsa-miR-7705 was involved in a tumor suppressor role in gastric cancer (**Figure 2**).

## Discussion

The occurrence of gastric cancer is a gradual process. Due to the influence of lifestyle, diet, genetic factors, and *Helicobacter pylori* infection, gastric cancer starts from gastritis and evolves into gastric ulcers and precancerous lesions, which may eventually lead to gastric cancer (1–3). At present, the gold standard for gastric cancer diagnosis is gastroscopy combined with pathological tissue biopsy. However, this is an invasive examination and is not suitable for large-scale screening. Therefore, the diagnosis of gastric cancer requires the support of new biomarkers. Since circRNAs covalently bind the 5’ and 3’ ends of RNAs into a circular structure (21), circRNAs are considered to be more stable than miRNAs, mRNAs, and long noncoding RNAs (22). Given that circRNAs are widely present in body fluids, including plasma, serum, exosomes, and urine, they are a new biomarkers for monitoring cancer development and progression (9, 10, 23, 24). To clarify the relationship between the abnormal expression of circRNAs and their prognostic value in gastric cancer patients, Chen et al. searched articles reporting the relationship between circRNAs and the prognosis of gastric cancer through a meta-analysis (25). Based on hazard ratios (HRs), they pointed out that two panels of five circRNAs (circ_0009910, hsa_circ_0000467, hsa_circ_0065149, hsa_circ_0081143, and circDLST; circSMARCA5, circLMTK2, hsa_circ_0001017, hsa_circ_0061276, and circ-KIAA1244) predicted a more considerable HR value. The results indicated that these two panels of dysregulated circRNAs might be considered as more suitable prognostic biomarkers in patients with gastric cancer (25). We noticed that hsa_circ_0061276 was one of the circRNAs. Therefore, we have a keen interest in exploring new circRNA-based diagnostic biomarkers of gastric cancer.

Since the levels of circRNAs in plasma are usually not very high (18), we utilized the more sensitive RT-ddPCR to detect hsa_circ_0061276 levels in plasma (26). Because of the current clinical limitations in the diagnosis of early gastric cancer, many patients miss the optimal treatment window (10). Therefore, we compared the difference in plasma hsa_circ_0061276 levels among healthy controls and patients with precancerous gastric lesions and early and advanced gastric cancer. We found that hsa_circ_0061276 has a certain diagnostic value for distinguishing healthy people from early gastric cancer (**Supplementary Figure 3**). The AUC was 0.6978, with a specificity as high as 90% and a negative predictive value as high as 80% (**Supplementary Table 3**). More importantly, plasma hsa_circ_0061276 has a certain value for distinguishing early and advanced gastric cancer. From these results, the copy number of plasma hsa_circ_0061276 had a gradually decreasing trend in these three groups (**Supplementary Figure 3**). We believe that hsa_circ_0061276 has the potential to be a biomarker for the diagnosis of gastric cancer.

To further study the role of hsa_circ_0061276 in the development of gastric cancer, cellular functional experiments were carried out. We found that overexpressed hsa_circ_0061276 inhibited the proliferation and migration of gastric cancer cells (**Figures 1A** and **2A**) and arrested the cell cycle at the G0/G1 phase (**Figure 3A**). Many studies have shown that circRNAs located in the cytoplasm can exert their biological functions by acting as miRNA sponges (27–29). We show that hsa_circ_0061276 is located in the cytoplasm (**Figure 4**), so hsa_circ_0061276 may play the role of miRNA sponge. Other studies have shown that low expression of circRNAs may lead to high expression of their associated miRNAs (30, 31). Combined with database screenings, three miRNAs were associated with hsa_circ_0061276 (**Supplementary Figure 5**). It was found that after overexpression of hsa-miR-6504-5p, hsa-miR-7705, or hsa-miR-7974 in gastric cancer cells, the expression of hsa_circ_0061276 was significantly decreased (**Figure 5**). Moreover, a double luciferase reporter gene assay confirmed that hsa_circ_0061276 had a binding effect on hsa-miR-7705 (**Figure 7A**). Therefore, hsa_circ_0061276 functions as a miRNA sponge. In a previous study, Hu et al. screened the three best independent prognostic miRNAs for bladder urothelial carcinoma, and hsa-miR-7705 was one of these prognostic miRNAs (32). Furthermore, they found that the survival time of patients expressing high hsa-miR-7705 levels was significantly shorter than that of patients expressing low levels (32). In addition, hsa-miR-7705 was upregulated in bladder urothelial carcinoma tissues (32), which was consistent with the upregulation in gastric cancer (**Figure 6**). However, the role of hsa-miR-7705 and other miRNAs including hsa-miR-6504-5p and hsa-miR-7705 in gastric cancer and the influence on downstream target genes are still unclear. Whether these circRNA–miRNA–mRNA axes regulate the occurrence and development of gastric cancer remains to be verified.

## Conclusions

In conclusion, we found that plasma hsa_circ_0061276 levels gradually declined throughout the course of gastric cancer progression. Overexpression of hsa_circ_0061276 suppressed the growth, migration, and cell cycle changes of gastric cancer cells, while downregulation of hsa_circ_0061276 demonstrated the opposite. Moreover, hsa_circ_0061276 had a miRNA sponge function. These findings indicate that hsa_circ_0061276 has a tumor suppressor role in gastric cancer.

## Data availability statement

The original contributions presented in the study are included in the article/**Supplementary Material**. Further inquiries can be directed to the corresponding author.

## Ethics statement

The studies involving human participants were reviewed and approved by Human Research Ethics Committee in Ningbo University (No. 2017022701). The patients/participants provided their written informed consent to participate in this study.

## Author contributions

YR, ZL, and YX collected clinical information; WS made the diagnoses; YR analyzed the data; YR and JG wrote the manuscript; and JG and YR designed the study. All authors contributed to the article and approved the submitted version.
