# Supplementary material for: Detecting plasma hsa_circ_0061276 in patients with gastric cancer by reverse transcription-digital polymerase chain reaction
Source: Front Oncol. 2022 Dec 23;12:1042248. doi: 10.3389/fonc.2022.1042248 (PMC9816570; doi:10.3389/fonc.2022.1042248)
Supplement: Supplementary file 1 [file DataSheet_1.docx]

**Supplementary data**

**Supplementary Table 1. Demographic characteristics of patients with early and advanced gastric cancer**

| Groups | *n* | Male | Female | Age (Mean ± standard deviation) |
| --- | --- | --- | --- | --- |
| Early gastric cancer | 30 | 23 | 7 | 64.63 ± 8.18 |
| Advanced gastric cancer | 78 | 57 | 21 | 66.85 ± 9.68 |

**Supplementary Table 2. The sequences of PCR primers**

| Name | Length (nt) | | Sequence (5′→3′) |
| --- | --- | --- | --- |
| Hsa_circ_0061276 forward primer  Hsa_circ_0061276 reverse primer  GAPDH forward primer  GAPDH reverse primer  NRIP1 forward primer  NRIP1 reverse primer  Hsa-miR-7705 forward primer  Hsa-miR-7705 reverse primer  U6 forward primer  U6 reverse primer | 24  24  20  20  23  24  22  24  25  19 | GGAGAAACACAGCCAGAAGGAAGT  CAGTTCTGAAATAGCTCACAATCC  AAGGTGAAGGTCGGAGTCAA  AATGAAGGGGTCATTGATGG  GAGCACTCCACCTTTACTTACAT  CAATCATACCTATCGGTTTATCTG  CGACCATGCAATAGCTCAGAAT  TATCCTTCTTCACGACTCCTTCAC  CAGCACATATACTAAAATTGGAACG  ACGAATTTGCGTGTCATCC | |

GAPDH, glyceraldehyde-3-phosphate dehydrogenase; NRIP1, nuclear receptor interacting protein 1.

**Supplementary Table 3. The diagnostic value of plasma hsa_circ_0061276 in the process of gastric cancer**

| Groups | AUC | Sensitivity (%) | Specificity (%) | Cut-off | PPV (%) | NPV (%) |
| --- | --- | --- | --- | --- | --- | --- |
| Healthy people *vs* Precancerous lesion | 0.6412 | 38.46 | 90.00 | 579.1 | 76.9 | 62.8 |
| Healthy people *vs* Early gastric cancer | 0.6978 | 46.67 | 90.00 | 579.1 | 60.9 | 83.5 |
| Healthy people *vs* Advanced gastric cancer | 0.7380 | 48.72 | 92.22 | 525.7 | 84.4 | 67.5 |
| Precancerous lesion *vs* Early gastric cancer | 0.5549 | 83.33 | 33.33 | 1562.0 | 32.5 | 83.9 |
| Precancerous lesion *vs* Advanced gastric cancer | 0.5902 | 69.23 | 47.44 | 1059.0 | 56.8 | 60.7 |
| Early gastric cancer *vs* Advanced gastric cancer | 0.5368 | 48.72 | 66.67 | 525.7 | 79.2 | 33.3 |

AUC, area under the receiver operating characteristic curve; PPV, Positive predictive value; NPV, Negative predictive value.


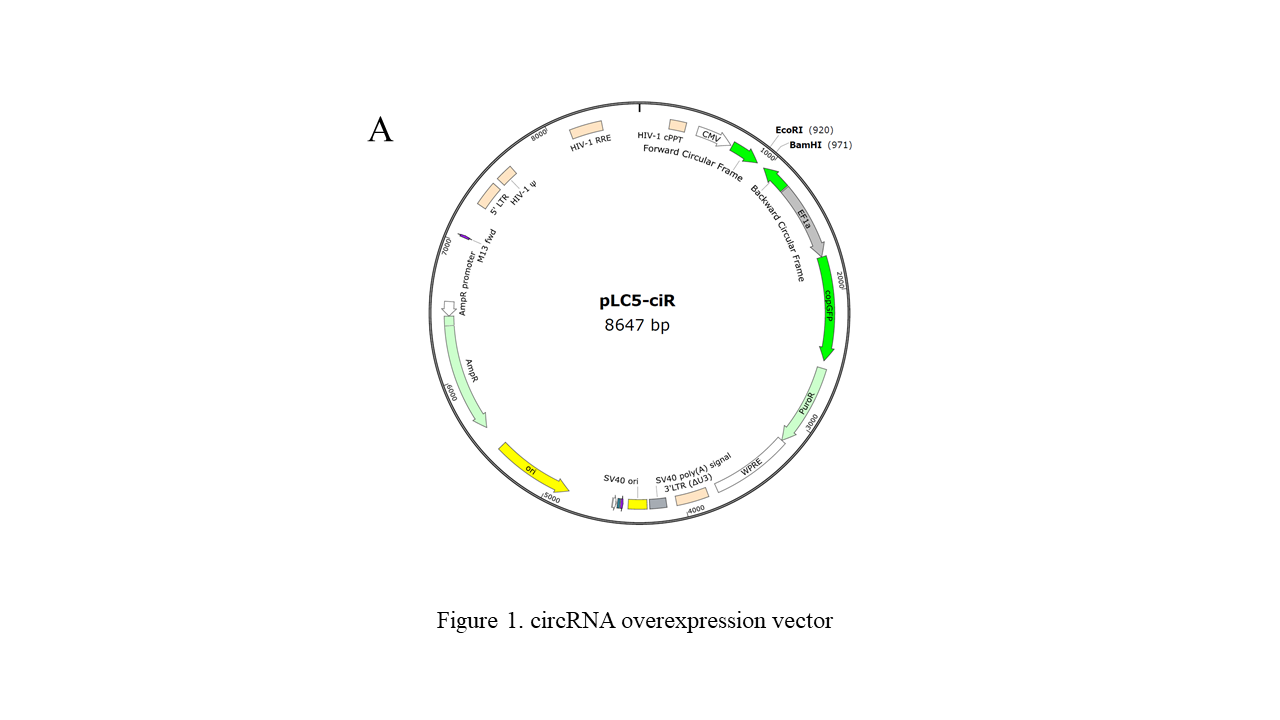

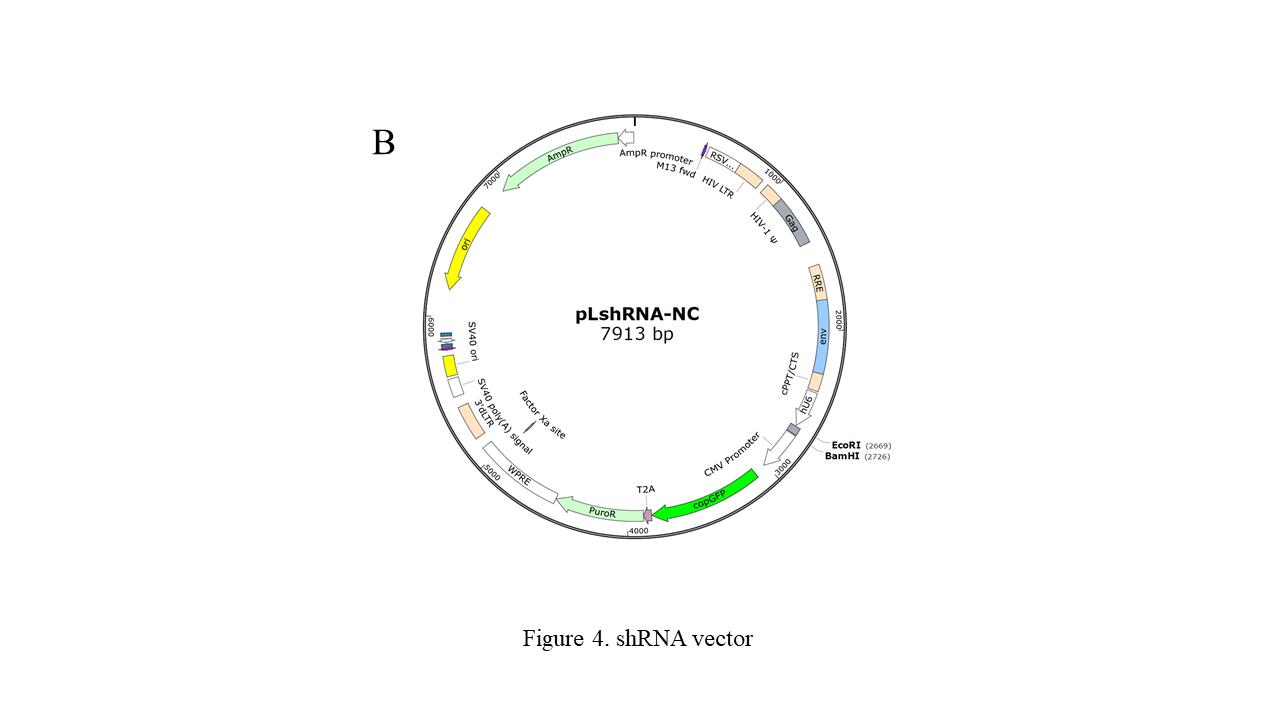


**Supplementary Figure 1. pLC5-ciR and pLshRNA vector structure provided by Geneseed (Guangzhou, China).**

**
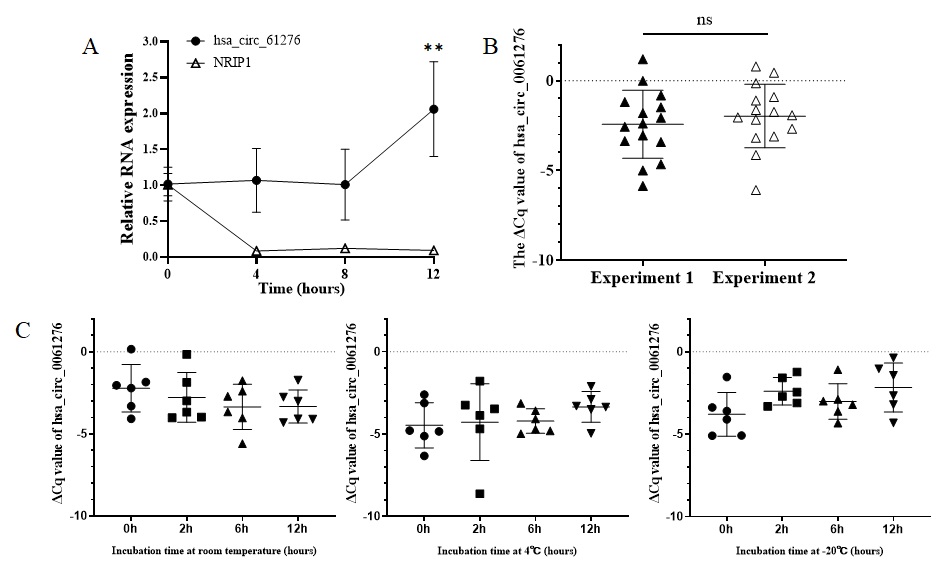
**

**Supplementary Figure 2. Results of studying on the stability of hsa_circ_0061276 in cells and plasma.** **(A)** Compare of the stability of hsa_circ_0061276 and NRIP1 mRNA in gastric cancer cells by actinomycin D treatment experiment. Independent samples *t*-test (two-tailed); ***P* < 0.01, *n* = 3. **(B)** Repeatability experiment of detecting plasma hsa_circ_0061276 in healthy people. Independent samples *t*-test (two-tailed); ns, no significance; *n* = 15. **(C)** Stability experiments of plasma hsa_circ_0061276 under several storage conditions. ANOVA; *P* > 0.05, *n* = 6.


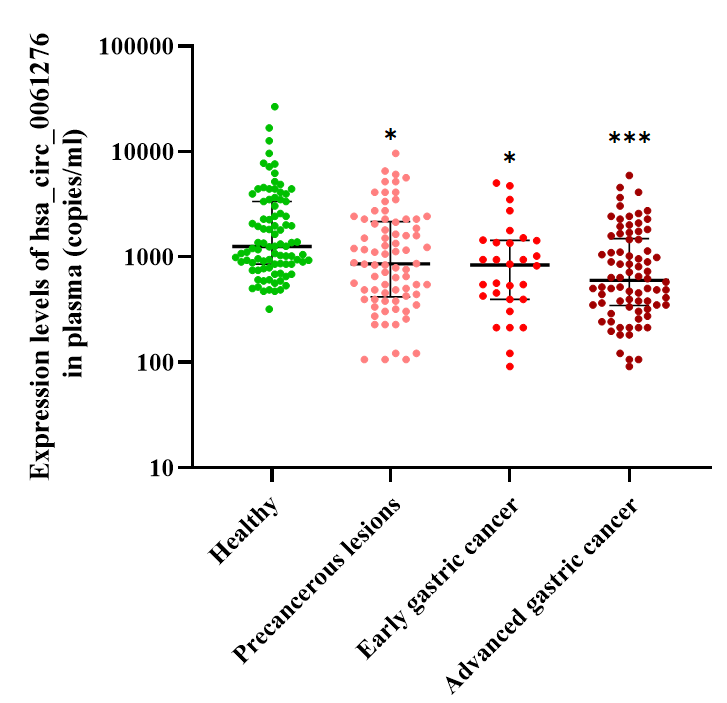


**Supplementary Figure 3. Plasma hsa_circ_0061276 levels in healthy people and patients with precancerous lesions, early and advanced gastric cancer.** Healthy people (*n* = 90), precancerous lesions (*n* = 78), early gastric cancer (*n* = 30), advanced gastric cancer (*n* = 78). ANOVA; **P* < 0.05, ****P* < 0.001.

**Supplementary Figure 4. Relative expression level of hsa_circ_0061276 in gastric cancer cells.**

Compared with GES-1, hsa_circ_0061276 was lowly expressed in two gastric cancer cells. Independent samples *t*-test (two-tailed); *****P* < 0.0001, *n* = 3.


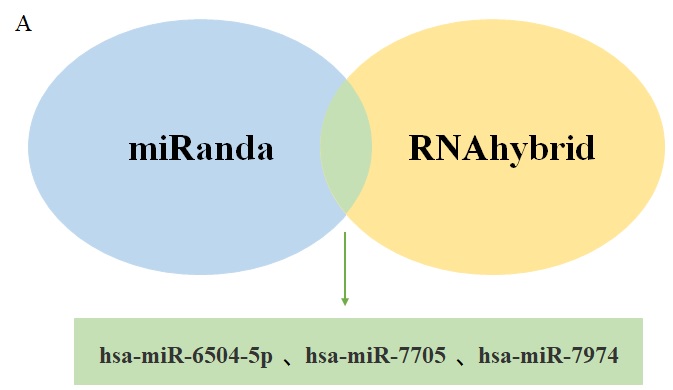


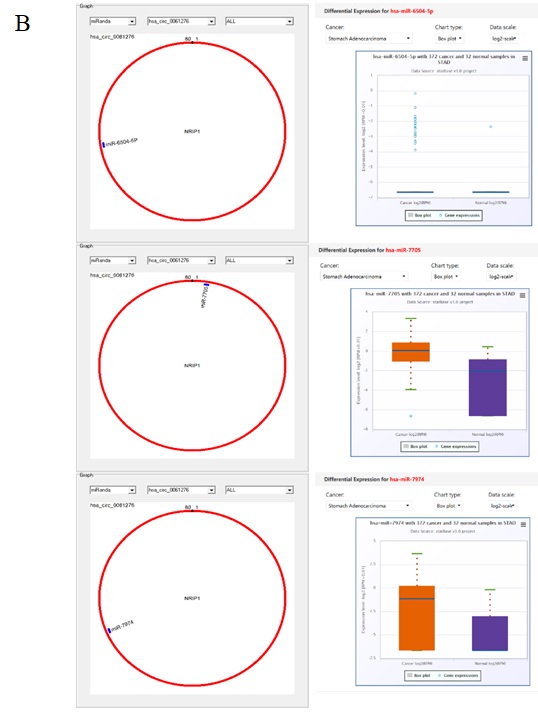


**Supplementary Figure** **5. The prediction of hsa_circ_0061276’s miRNA sponge function and the corresponding miRNAs’ expression analysis.** **(A)** Predicting miRNAs that may bind with hsa_circ_0061276 by miRanda and RNAhybrid database. **(B)** Predicting the binding site between hsa_circ_0061276 and miRNAs, and miRNAs’ differential expression between gastric cancer tissues and corresponding normal tissues through ENCORI database.
